# PACVr: plastome assembly coverage visualization in R

**DOI:** 10.1186/s12859-020-3475-0

**Published:** 2020-05-24

**Authors:** Michael Gruenstaeudl, Nils Jenke

**Affiliations:** 1 Institut für Biologie, Systematische Botanik und Pflanzengeographie, Freie Universität Berlin, Berlin, 14195 Germany; 2 Institut für Bioinformatik, Freie Universität Berlin, Berlin, 14195 Germany

**Keywords:** software, R package, genome assembly, plastid genome, sequencing coverage, visualization

## Abstract

**Background:**

Plastid genomes typically display a circular, quadripartite structure with two inverted repeat regions, which challenges automatic assembly procedures. The correct assembly of plastid genomes is a prerequisite for the validity of subsequent analyses on genome structure and evolution. The average coverage depth of a genome assembly is often used as an indicator of assembly quality. Visualizing coverage depth across a draft genome is a critical step, which allows users to inspect the quality of the assembly and, where applicable, identify regions of reduced assembly confidence. Despite the interplay between genome structure and assembly quality, no contemporary, user-friendly software tool can visualize the coverage depth of a plastid genome assembly while taking its quadripartite genome structure into account. A software tool is needed that fills this void.

**Results:**

We introduce ’PACVr’, an R package that visualizes the coverage depth of a plastid genome assembly in relation to the circular, quadripartite structure of the genome as well as the individual plastome genes. By using a variable window approach, the tool allows visualizations on different calculation scales. It also confirms sequence equality of, as well as visualizes gene synteny between, the inverted repeat regions of the input genome. As a tool for plastid genomics, PACVr provides the functionality to identify regions of coverage depth above or below user-defined threshold values and helps to identify non-identical IR regions. To allow easy integration into bioinformatic workflows, PACVr can be invoked from a Unix shell, facilitating its use in automated quality control. We illustrate the application of PACVr on four empirical datasets and compare visualizations generated by PACVr with those of alternative software tools.

**Conclusions:**

PACVr provides a user-friendly tool to visualize (a) the coverage depth of a plastid genome assembly on a circular, quadripartite plastome map and in relation to individual plastome genes, and (b) gene synteny across the inverted repeat regions. It contributes to optimizing plastid genome assemblies and increasing the reliability of publicly available plastome sequences. The software, example datasets, technical documentation, and a tutorial are available with the package at https://cran.r-project.org/package=PACVr.

## Background

The sequencing and comparison of complete plastid genomes has become a popular method in plant evolutionary research, rendering the precise genome assembly and its quality assessment of high importance. The plastid genomes of most photosynthetically active land plants display a circular, quadripartite structure and comprise two single copy (SC) regions separated by two identical inverted repeats (IR) [1]. A total of four partitions with markedly different lengths can, thus, be defined in typical land plant plastomes: the large single copy (LSC) region of ca. 70-90 kilobases (kb), the small single copy (SSC) region of ca. 15-25 kb, and the two IR regions (IRa and IRb) of ca. 20-25 kb each [2]. The IR regions represent reverse complements of each other and are primarily homogenized through a recombination-mediated replication process [3, 4]. The plastid genomes of most photosynthetically active land plants encode a total of ca. 100-120 proteins, which play a central role in organelle metabolism and photosynthesis [5]. Due to their strong structural conservation, uniparental inheritance, a near absence of recombination, and a high copy number per plant cell, plastid genomes are highly suitable for comparative genomic studies [6]. Numerous investigations have sequenced and compared complete plastid genome sequences over the past decade [7, 8], and the number of publicly available plastid genomes continues to increase dramatically [9]. Recent studies on plastid genome structure and evolution have evaluated polymorphisms across hundreds [10–13] or even thousands [14, 15] of plastid genome sequences, rendering the precise assembly process of plastid genomes and their quality assessment ever more important.

Despite the development of assembly algorithms customized for plastid genomes, the plastome assembly process remains imperfect and often requires the verification, if not manual correction, of the assembly product. Concurrent with the surge in plastid genome sequencing, many new algorithms and pipelines specifically designed for the assembly of plastid genomes have been developed [16–23]. Most of these tools allow a more accurate and targeted assembly of the plastid genome than generic assembly software, but in many cases some form of manual intervention or post-processing of the assembly results remains necessary [18, 21]. The post-processing of automated assembly results often pertains to the correction of the IR length [6, 9], differences in junction boundaries [24], and genome circularization [25]. Common uncertainties and outright errors in plastid genome assemblies include the inequality of the IR regions in length or sequence [26–28], long homopolymer runs [9, 29], and the imperfect duplication of repeats at the junction of the SC regions [24]. The differential orientation of the SSC, by contrast, does not constitute an assembly error but reflects the natural presence of heteroplasmy in organelle genomes [30, 31]. To ensure correctness and reproducibility in plastid genome sequencing and analysis, it is paramount to confirm the validity of plastid genome assemblies [9, 21, 24]. Many of the ambiguities and putative errors recognized in published plastid genome sequences, including those of genome annotations [9, 27, 28], could potentially be averted by the application of simple quality assessment strategies [21, 29].

Several measures have been used to indicate the quality of plastid genome assemblies, including contiguity metrics and the length and sequence equality of the IR regions, but sequencing coverage remains one of the most popular proxies for assembly quality. In genome research, the length of the shortest among all those contigs that cover at least 50% of a reference genome is often used as an indicator for the quality of a draft genome [32]. The closer this length is to the complete length of the reference genome, the more confidence is placed in the completeness and, by extension, the quality of the assembly [33]. This concept is one of several contiguity metrics used to indicate the quality of a genome assembly (e.g., NG50, [32]; NA50 and NGA50, [34]). However, these contiguity metrics are difficult to apply to so far unsequenced organisms due to the requirement of a known reference sequence. Another, more specific measure for validating the quality of genome assemblies constitutes the degree of gene synteny across draft genomes or subsections thereof [35]. The IRs of a plastid genome, for example, represent recombinogenic isomers and, thus, share the same DNA sequence and gene synteny [3, 36]; exceptions to this rule are very rare [37]. Equality in length, sequence, and gene synteny of the IR regions can, thus, be used as a general indicator for the quality of the plastid genome assembly [27]. The depth of sequencing coverage (’coverage depth’ hereafter) represents yet another indicator for the quality of a genome assembly [38]. Average coverage depth is defined as the average number of times each nucleotide of a genome region is represented by aligned reads from a sequence set [39]; it is a unit-less integer. Coverage depth is an important and highly popular indicator for the quality of a genome assembly in biological research [40, 41]. For plastid genomes, coverage depth is reported almost by default in relation to genome assemblies [6, 42] and has been implemented as a quality metric in several plastome assembly pipelines [18–20]. Information on coverage depth is critical for the assessment of large-scale sequence rearrangements or other structural variation of a genome because a greater coverage depth increases the chance that rearrangement endpoints are captured and confirmed by multiple independent reads [39, 43]. Information on coverage depth is also critical for the assembly process itself, as many de novo assembly algorithms operate under the implicit assumption of even coverage depth across the target genome [6, 44, 45]. In the present investigation, coverage depth, as well as gene synteny across the IR regions, are used as specific indicators for plastid genome assembly quality.

**Fig. 1:**
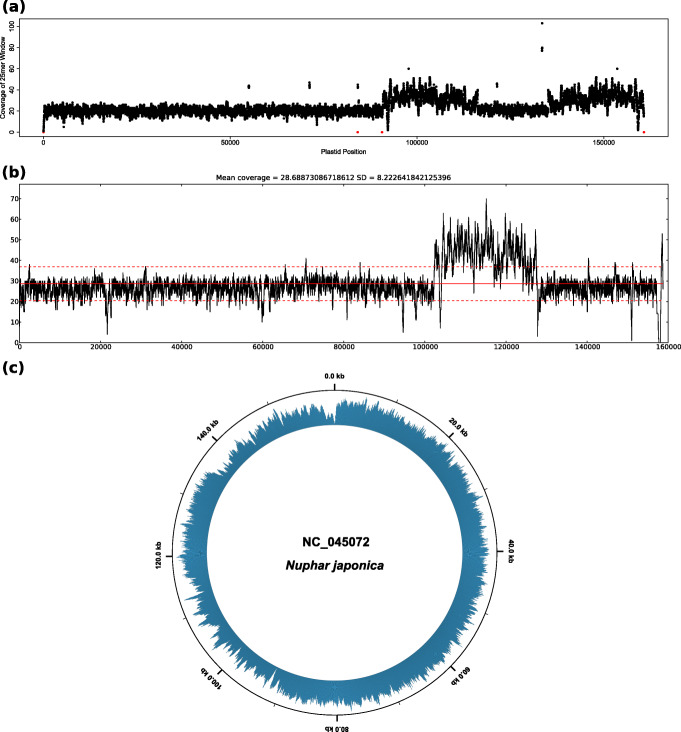
Visualizations of coverage depth of the plastid genome of *Nuphar japonica* (GenBank accession NC_045072) as generated by the software tools (**a**) FastPlast, (**b**) IOGA, and (**c**) Circleator. For easier viewing, the size of the individual data points was reduced in the visualization of FastPlast, and the tick mark at 160 kb was removed in the visualization of Circleator

Currently available software tools can generate either unpartitioned plots of plastome coverage depth or quadripartite plastome maps, but the simultaneous, user-friendly visualization of both aspects is presently unsupported. When employing currently available software tools, plant biologists must decide if they wish to visualize either plastome sequencing coverage as unpartitioned, often linear plots or, alternatively, the circular, quadripartite structure of a plastid genome. The assembly pipeline FastPlast [19], for example, analyzes coverage depth during run-time and, upon genome assembly, generates a linear coverage plot as part of the pipeline execution (Fig. 1a). Similarly, the assembly pipeline IOGA [46] generates linear coverage plots during run-time, allowing users to evaluate the progress of the assembly process during different pipeline iterations (Fig. 1b). The assembly pipeline ORG.asm [20] also estimates average coverage depth during the assembly process but does not visualize this metric. None of these assembly pipelines generate visualizations that account for the circular, quadripartite structure of the plastid genome or for the location of the individual plastome genes. On the other hand, several software tools and web-services exist that visualize complete plastid or bacterial genomes as circular maps. The web-service OrganellarGenomeDraw (OGDRAW; [47, 48]), for example, generates circular maps of plastid and mitochondrial genomes and visualizes gene position and GC content across the genomes. Similarly, the software Circleator [49] generates circular maps of bacterial genomes and can visualize gene position, GC content, and single nucleotide polymorphism locations in comparison to a reference genome. When co-supplied with text-based configuration instructions and a read mapping file, Circleator can also visualize coverage depth on the circular visualizations (Fig. 1c), but the configuration instructions are complex, and unless an intricate, multi-layered visualization procedure is applied, additional genome annotations such as genes are not displayed. The software Circos [50] can also be used to generate elaborate visualizations of circular genomes, including plastomes [51–53], but even more bioinformatics expertise is required to generate the source code underlying these visualizations, which is typically beyond the ability of a normal user in plant biology. Several older software tools and web-services for generating circular genome maps also exist [54–58], but their application in recent research has been minimal, and some of these services have become inaccessible (e.g., [54, 57, 58]; inaccessible since at least October 2018). To the best of our knowledge, none of the presently available software tools can visualize the coverage depth of a plastid genome assembly on a circular, quadripartite plastome map, as well as gene synteny across the IR regions, while simultaneously displaying the locations of the plastome genes and the location and relative sizes of the SC and the IR regions, especially in a user-friendly fashion.

Given the plethora of complete plastid genomes generated in biological research each year [9], strong demand for a software tool exists that enables a visual quality assessment of plastid genome assemblies. Specifically, it would be desirable to have a tool that allows users to visually explore the coverage depth of a plastid genome assembly as well as the gene synteny across its IR regions [59], as both aspects are indicative for the quality of the genome assembly. To be useful to a wide audience, such a software tool must fulfill four criteria: it must (a) be user-friendly and applicable to users with minimal bioinformatics knowledge; (b) generate publication-ready visualizations that allow the determination if and where a genome assembly displays insufficient coverage depth; (c) allow an easy integration into automated workflows or analysis pipelines; and (d) allow users to set customized window sizes and thresholds for coverage depth calculation. Here, we present such a tool, titled ’PACVr’ for ’Plastome Assembly Coverage Visualization in R’. PACVr is a package for the common statistical environment R [60] that visualizes (i) coverage depth of a plastid genome assembly on a circular, quadripartite plastome map, and (ii) gene synteny across the IR regions of the genome assembly. Specifically, PACVr visualizes coverage depth across the entire plastid genome in user-defined window sizes and in relation to the gene annotations, calculates and displays average coverage depth values for each of the four plastome regions, highlights sectors with coverage depth below a user-specified threshold, and visually connects the genes of one IR with their counterparts of the other IR using variable-width connector lines. By applying PACVr upon plastid genome assembly, users can visually inspect coverage depth and IR gene synteny across the input genome and, where applicable, identify regions of potentially reduced assembly confidence. Specifically, users can identify sectors of a plastid genome with low coverage depth or IRs with missing gene synteny and then subject these sectors to re-evaluation or post-processing. Upon presenting the details of the software, we illustrate the application of PACVr on four plastid genome assemblies from different plant lineages. Two of the assemblies represent newly sequenced plastid genomes with a plastome size typical for most angiosperms and a quadripartite genome structure; for these assemblies, we compare the visualizations of PACVr with the output of other software tools for visualizing plastome coverage depth. The other two assemblies represent previously published plastid genomes with plastome sizes that considerably deviate from the typical size range of plastid genomes; one of these assemblies also represents a plastid genome without quadripartite genome structure. Our application of PACVr on empirical data, thus, illustrates the flexibility of the software with regard to plastid genome size and structural configuration.

## Implementation

### Input and output specifications

The input to PACVr consists of two different files of common file format which contain information on (A) genome sequence and structure, and (B) coverage depth. Information on genome sequence and structure, as well as the genes encoded by the sequence, is supplied via an input file in the GenBank flatfile format. GenBank flatfiles represent the default file type for sequence retrievals from NCBI Nucleotide [61] and contain one or more sequence records, with each record comprising general metadata, an annotation table with the names and locations of genes and other sequence features, and the nucleotide sequence itself [62]. In PACVr, GenBank flatfiles are parsed via the R package genbankr [63] and must, thus, contain only a single sequence record per file, with the locus name no longer than ten alphanumeric characters. Moreover, genbankr requires the location of sequence features that span multiple positions or occur on complementary strands to be specified with the use of only a single invocation of the commands ’join’ and ’complement’ each, and all sequence features of class ’exon’ to be removed. For optimal visualizations, the sequence record of the GenBank file should represent a complete, fully annotated plastid genome and contain feature annotations for each of the two IRs, if these repeats are naturally present in the genome; flatfile qualifiers for the IRs must hereby have the text values ’IRa’ and ’IRb’, or ’inverted repeat A’ and ’inverted repeat B’, respectively. For optimal visualizations, the sequence record should have a total sequence length between 50 kb and 250 kb. This preferred size range encompasses all plastid genomes of photoautotrophic land plants currently available on GenBank (sizes of the smallest and largest photoautotrophic embryophyte plastome on GenBank as of 01 January 2020: 59,190 bp [NC_014874] and 242,575 bp [NC_031206], respectively) and is a consequence of the practical limitations of scaling a circular, multi-layered plastome map to overall genome size. The scaling conducted by PACVr particularly aims to balance the visualization of the complete genome with sufficient spacing between adjacent plot layers and a font size large enough for text elements to be legible. Information on coverage depth is supplied via an input file in the binary alignment/map (BAM) format, which stores alignment and mapping information [64] and is typically generated by the mapping of sequence reads to a reference genome with short read alignment packages [65] (such as BWA [66] or Bowtie2 [67] in conjunction with the software samtools [64]). To be suitable for PACVr, the BAM file must also be indexed and, thus, accompanied by an ancillary index file. Generating the BAM file is done prior to, and independent of, the functionality of PACVr and can be conducted under a series of different settings, which may be reflected in the resulting visualization. For example, users may wish to visualize coverage depth calculated only from sequence reads that map to the reference genome as concordant read pairs, which can be beneficial in the identification of assembly errors [68]. Similarly, users may wish to visualize coverage depth calculated only from sequence reads that map to more than one location in the reference genome, which, if applied to plastid genomes, typically highlights the location of the IRs. This autonomy in generating BAM files provides users with considerable flexibility in the application of PACVr, especially as part of a bioinformatic workflow. Several additional input parameters can be specified upon initiation of PACVr, including the window size used for calculating coverage depth, the threshold below which coverage depth is highlighted, and the name of the output file, among other aspects, but these parameters are optional and have well-tested default values set for them.

The output of PACVr is a multi-layered, annotated plastome map with coverage information across the genome. Specifically, PACVr generates a circular, quadripartite map of the plastid genome in which coverage depth values are displayed as histogram bars, with bars below a predefined threshold highlighted in red and partition-wide average coverage values superimposed as horizontal, yellow lines. The map also displays positional information in regular intervals in the form of labeled tick marks as well as the location of all plastome genes, allowing the user to relate areas of low coverage depth to specific genome regions and genes. The map generated by PACVr is saved in PDF format to a user-defined output file.

### Coverage calculation and display

Coverage depth is calculated by PACVr via the application of user-defined window sizes with the software mosdepth [69]. Window-based coverage calculations have the flexibility of measuring coverage on customized scales, which can be necessary to account for the variability in read length across different Illumina reagent types or sequencing cycle numbers. Using a sorted BAM file plus its ancillary index file as input, mosdepth rapidly infers the coverage of a particular chromosome by tracking all start and end positions of mapped sequence reads and calculating the cumulative sum of their incremented start positions while decrementing the respective end positions [69]. Based on the results of mosdepth, PACVr infers the average coverage depth for each of the four regions of the plastid genome (i.e., LSC, SSC, IRa and IRb). Two types of coverage depth information are plotted on the plastome map: (i) window-based depth values are displayed in the form of a circular histogram, with the width of each histogram bar equal to the width of the window size, and (ii) partition-wide coverage averages are displayed as horizontal, yellow lines superimposed on the histogram bars as well as numerically in the plastome map legend. PACVr is, thus, different from most other software tools for visualizing coverage depth, which typically display coverage depth as stacked sequence reads [70, 71], line graphs [46], dot graphs [19] or bedGraphs plots [72], and primarily on linear representations of the input genome.

### IR equality assessment and display

Equality among the IR regions is evaluated by PACVr both directly and indirectly. The direct evaluation is done by the computational comparison of the sequences of IRa and IRb, the indirect evaluation via the numerical and visual comparison of number, length, and location of all genes contained in the IRs. Specifically, the software conducts a two-step procedure in which equality in sequence, sequence length, and the number of genes is confirmed across the two IR regions, and the equality then visualized by connecting the matching genes of the two IRs via blue connector lines. To that end, PACVr computationally extracts the two IR regions from the input sequence record, stores their sequences as well as the names, start and end positions of all IR genes in separate data frames, and compares the exact number, length, and location of the genes across both regions. Any difference in sequence, sequence length, or gene complement between the two IRs results in a warning message to the user. PACVr then visualizes the equality between the IRs by connecting genes with identical names across the regions using blue connector lines. The lines hereby originate and end at the central nucleotide of each gene shared between the two IRs. The start and end width of these connector lines can be set to be uniform or proportional to the length of the genes they connect. Any difference in name or location of the IR genes becomes visible through unequal or missing connector lines, thus enabling the visual assessment of equality among the IR regions regarding gene presence and synteny. This visualization of gene location and synteny contributes to the discovery of rules and patterns in genome orientation and rearrangements [73].

### Visualization

PACVr employs RCircos [74] as the visualization engine. RCircos is an R implementation of the Circos environment [50] and is employed by PACVr to visualize the various aspects of plastome structure and coverage depth in four separate layers. In the first, outermost layer, PACVr displays length-labeled tick marks at each decile of total genome length to provide positional information across the genome. The layer also plots the names and relative positions of the individual regions of the quadripartite genome structure (i.e., LSC, SSC, IRa and IRb), with each region marked in a different color for easier delineation. If none or only one of the IRs are detected in the input genome, this layer displays a homogeneous color. In the second layer, PACVr plots the names and positions of all genes of the plastid genome, with gene positions indicated by their central nucleotide. In the third layer, PACVr plots the coverage depth of the plastid genome in the form of a circular histogram, with bars displaying one of two possible colors depending on their depth value relative to a user-defined threshold: bars with a coverage depth above the threshold are displayed in black, bars below the threshold in red. The threshold is by default specified relative to the average genome-wide coverage depth, but can optionally be set as an absolute value. Moreover, this layer indicates the average coverage depth of each of the four plastome regions via a horizontal, yellow line, which is missing in areas without coverage. In the fourth, innermost layer, PACVr plots blue connector lines that connect genes with identical names across the two IR regions, with lines originating and ending at the central nucleotide of each gene. At the lower left of the circular graph, PACVr prints a legend that displays the absolute and relative coverage depth threshold values below which histogram bars are highlighted, as well as the numeric values of average coverage depth of the four plastome regions. The name of the organism under study, which is parsed from the GenBank input file, is displayed as the figure title.

### Accounting for quadripartite structure

The quadripartite structure of plastid genomes requires adjustments in the calculation of coverage depth and the visualization of IR equality compared to unpartitioned chromosomes. By default, PACVr calculates window-sized coverage depth values and, based on these, the region-wide average for each of the four plastome regions. However, PACVr would double-count the coverage of those windows that span across a region boundary, unless the coverage calculation included a special adjustment. Similarly, PACVr requires customization when visualizing the equality of gene position and synteny between the two IR regions. Natural expansions in IR size can cause genes located near the border of a single copy and an IR region to be displaced from one region into the other over time [75, 76]. Without a customized visualization, genes that are located primarily in the single copy region but span into the IR (or vice versa) would not be included in the IR equality visualization if the central nucleotide of genes used to connect the counterparts was located outside the IR. This can be particularly problematic with large plastome genes such as *ycf1* and *ycf2*, which are located near the 5’ end of the SSC and the IRa, respectively, in most angiosperms and represent nearly 10% of the unit-genome length [77]. A similar issue would arise with trans-spliced plastome genes whose exons were located in an SC and an IR region, respectively (e.g., *rps12* in many angiosperms [78]). Thus, the code of PACVr was customized to split genes that span more than one genome region into two separate parts along the region boundary and to treat both parts as separate units. PACVr tracks the position of these unequal units in relation to the region boundaries throughout software execution and corrects the location of the original genes and, by extension, their gene labels and histogram bars during the generation of the final plastome map using a size correction factor.

### Installation, dependencies and usage

PACVr was written in R and can be installed via the Comprehensive R Archive Network (CRAN; https://cran.r-project.org/) using the R command install.packages(^′^*PACVr*^′^). It requires the presence of the R packages optparse [79], genbankr [63], and RCircos [74] as dependencies and employs several generic library functions developed for high-throughput genomic analysis [80]. Additionally, PACVr requires the software mosdepth [69] to be present on the system, which can be installed via the Unix shell command conda install mosdepth. The source code of PACVr is available via Github at https://github.com/michaelgruenstaeudl/PACVr. The technical documentation and a user tutorial (vignette) is distributed as part of the R package. The vignette provides example commands for the installation and execution of PACVr as well as for the generation of BAM files under different read filtering settings.

Two mandatory and nine optional input parameters can be specified when invoking PACVr. The mandatory input parameters are: the name of, and file path to, the input GenBank file, and the name of, and file path to, a sorted and indexed BAM file. The optional input parameters are: (i) the window size for calculating coverage depth, with a default value of 250; (ii) the shell command to execute mosdepth, with a default command of mosdepth; (iii) the coverage depth threshold above which histogram bars are plotted in red as opposed to the default black, with a default value of 0.5; (iv) the selection if the threshold value is specified relative to the average genome-wide coverage depth as opposed to representing an absolute value, with the default set to true; (v) the selection if the coverage depth values are to be log-transformed prior to visualization, with the default set to false; (vi) the selection if and what type of connector lines to draw between matching genes of the IRs, with the default line type displaying a start and end width proportional to the length of the genes that the lines connect; (vii) the size of all text elements of the resulting visualization relative to the maximum font size, with the default set to 0.5; (viii) the decision to remove all temporary files generated during the coverage depth calculation, with the default set to true; and (ix) the name of, and file path to, the output file, with the output saved as ./PACVr_output.pdf by default. The software can be invoked either from within the R environment or directly from a Unix shell. A complete list of the short- and long-flag command-line (CLI) arguments available when invoking PACVr from the Unix shell is displayed via the shell command Rscript./inst/extdata/PACVr_Rscript.R -h/—help. In the framework of an automated workflow (and upon setting the location of PACVr to a shell variable with the same name), the following shell command can, for example, be used to execute PACVr on the empirical dataset co-supplied with the R package:


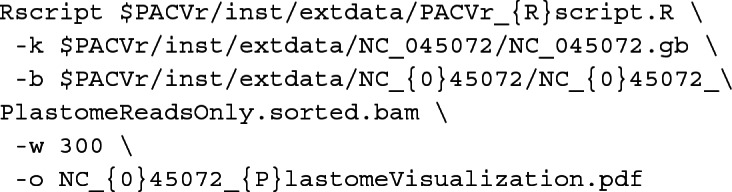


### Testing of software

To evaluate and demonstrate the functionality of PACVr, the software was tested under a variety of different settings. First, PACVr was tested on empirical data of four complete plastid genomes. Specifically, the software was employed for visualizing coverage depth and IR equality of the assemblies of two novel as well as two previously published plastid genomes. The novel plastid genomes represent the angiosperm species *Archidasyphyllum excelsum* (Asteraceae) and *Nuphar japonica* (Nymphaeaceae) and display a quadripartite genome structure as well as a genome size typical for the majority of angiosperms [2]. The previously published plastid genomes represent the angiosperm species *Pelargonium* x *hortorum* (Geraniaceae; [81]) and the non-photosynthetic green algae *Prototheca cutis* (Chlorellaceae; [82]) and display a genome size that substantially deviates from the typical size range of angiosperm [2] and green algae plastomes [83], respectively. Moreover, the plastid genome of *Prototheca cutis* naturally lacks the IR regions and, thus, a quadripartite genome structure [82, 84]. Details on the length and position of the different plastome regions present, the overall size of the genome, the GenBank accession number, and, in case of previous publication, the accession number of the original sequence reads are given in Table 1. The plastid genomes of *Archidasyphyllum excelsum* and *Nuphar japonica* were generated for this investigation via Illumina MiSeq sequencing following the sequencing protocol of [27] and the assembly workflow of [21]; the plastid genomes of *Pelargonium* x *hortorum* and *Prototheca cutis* were downloaded from GenBank. Information on coverage depth was generated for each plastid genome by mapping the original sequence reads to the complete genome sequence using BWA and samtools, which resulted in one sorted and indexed BAM file per genome. For each of the newly generated plastid genomes, spikes in the coverage depth were capped at a maximum of 20x to keep the size of the BAM files at a maximum of 2.5 megabytes per file and, thus, ensure a lightweight distribution of the R package once these BAM files were included in the package as example data. The cap was administered via script ’bbnorm.sh’ of the software BBTools v.33.89 [85], which removes spikes in sequence coverage via a stochastic normalization procedure. Upon preparation of all input files, PACVr was employed on each of the four plastid genomes using default parameter values. Second, PACVr was tested on five different operating systems. Specifically, we tested the software on macOS 10.13.6 (High Sierra), macOS 10.14.6 (Mojave), Arch Linux 4.18, Debian 9.9, and Ubuntu 18.10. Under each system, PACVr was invoked both from within the R environment as well as directly from a Unix shell. Third, PACVr was compared to three software tools that are capable of visualizing plastome sequencing coverage. Specifically, we employed the tools Circleator v.1.0.2, FastPlast v.1.2.8, and IOGA v.20160910 to visualize the coverage depth of the two newly generated plastid genomes and compared their output to the default visualizations of PACVr.

**Table 1:** The GenBank and the sequence read archive (SRA) study accession numbers as well as details on genome size and structure of the four plastid genomes used to demonstrate the functionality of PACVr. All size and position values are given in bp. Abbreviations used: n.a. = not applicable; pos. = position

Species name	GenBank	SRA study	Total size	Pos. LSC	Pos. (size) IRb	Pos. (size) SSC	Pos. (size) IRa
*Archidasyphyllum excelsum*	MH899017	SRP253816	151,880	1..83,242	83,243..108,250 (25,007)	108,251..126,872 (18,621)	126,873..151,880 (25,007)
*Nuphar japonica*	NC_045072	SRP253784	160,761	1..90,653	90,654..116,283 (25,629)	116,284..135,131 (18,847)	135,132..160,761 (25,629)
*Pelargonium* x *hortorum*	NC_008454	SRP015898	217,942	1..59,710	59,711..135,457 (75,746)	135,458..142,209 (6,751)	142,210..217,942 (75,732)
*Prototheca cutis*	NC_037480	DRP002975	51,673	n.a.	n.a.	n.a.	n.a.

## Results

### Visualizations by PACVr

**Fig. 2:**
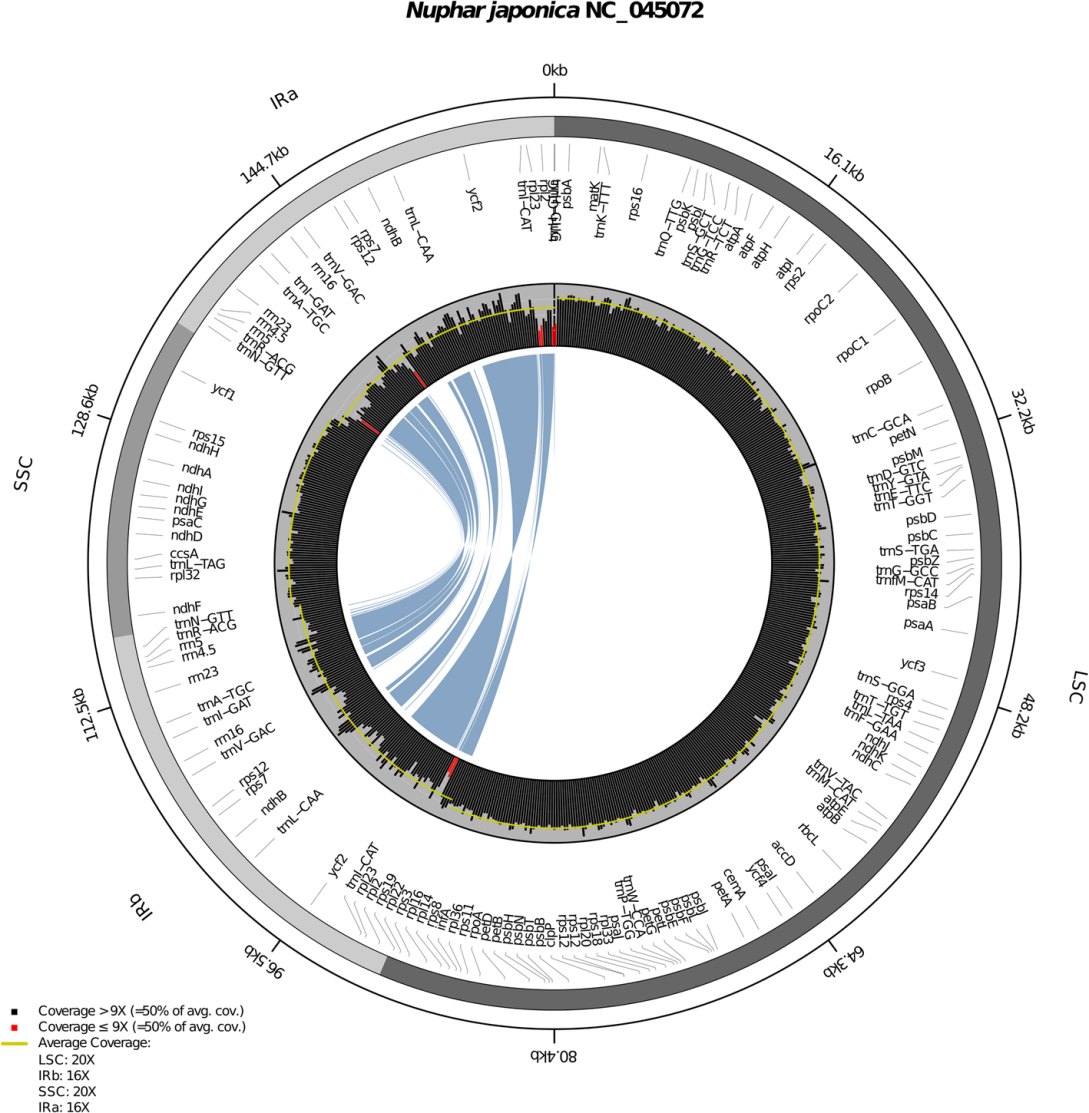
Visualization of coverage depth and IR equality of the plastid genome of *Nuphar japonica* as generated via PACVr

PACVr was successfully applied in the visualization of coverage depth and IR equality of the four complete plastid genomes used for evaluating the functionality of the software. Specifically, PACVr visualized the coverage depth of each plastid genome in relation to its circular, often quadripartite genome structure and illustrated the equality of its IR regions regarding gene position and synteny (if such regions existed in the genome). Based on the resulting visualizations, several important observations were made. First, the visualizations indicated differences in region-wide average coverage depth and the presence of genomic areas with markedly lower coverage depth compared to other areas of the same genome in each of the plastid genomes under study. In the plastid genome of *Nuphar japonica* (Fig. 2), for example, the average coverage depth of both IRs was detected to be 16x compared to 20x for the LSC and the SSC, respectively. Moreover, a window-sized coverage depth below 50% of the average genome-wide coverage depth was identified in several locations of the IRs, particularly at the 5’ end of the IRb and, conversely, the 3’ end of the IRa (assuming a single reading direction for the entire genome), which corresponds to the location of the ribosomal protein genes *rpl2* and *rpl23*. In the plastid genome of *Archidasyphyllum excelsum* (Additional file 1: Figure S2), the average coverage depth of the IRs was also detected to be lower than that of the LSC or the SSC. Moreover, a window-sized coverage depth below 50% of the average genome-wide coverage depth was identified in several locations of the IRs, particularly at or near the 5’ end of the IRb (and, conversely, the 3’ end of IRa), which corresponds to the location of gene *ycf2*; a suboptimal coverage depth was also detected in one calculation window of the LSC (near *trnD*-GUC, a gene encoding one of the transfer RNAs for aspartate). Successful visualizations of coverage depth were also conducted for the plastid genomes of *Pelargonium* x *hortorum* (Additional file 2: Figure S3) and *Prototheca cutis* (Additional file 2: Figure S4), despite their substantial deviations in genome size from the typical size range of angiosperm and green algae plastomes. The genome-wide average coverage depth of these genomes was calculated to be 4,569x and 615x, respectively, but could not be inferred by region, as the IR annotations of each genome were either unequal in length (*Pelargonium* x *hortorum*; Table 1) or missing to reflect the natural state (*Prototheca cutis*). The threshold value for highlighting histogram bars was set to 100% of the average genome-wide coverage depth for both genomes to contrast this option with the visualizations of the newly generated plastid genomes. Second, the visualizations by PACVr indicated strong gene synteny across the IRs of those plastid genomes under study that possess a quadripartite genome structure. Specifically, the symmetric display of equal gene position and length via blue, variable-width connector lines between the IRs of a genome indicated IR gene synteny in *Nuphar japonica*, *Archidasyphyllum excelsum*, and *Pelargonium* x *hortorum* (Figs. 2, S2, and S3). By contrast, PACVr automatically skipped the visualization of IR gene synteny for the plastid genome of *Prototheca cutis*, as this non-photosynthetic green alga does not possess IRs in its plastid genome or, by extension, the relevant IR feature annotations in its sequence record, rendering an evaluation and visualization of IR gene synteny obsolete. In summary, the IR regions of those plastid genomes with a quadripartite genome structure were found to display areas of reduced coverage depth, but equality in sequence length and gene position and, by extension, the presence of gene synteny between the IRs. Identical visualizations were retrieved when executing PACVr on macOS or Linux, confirming the compatibility of PACVr to different operating systems.

### Comparison to other software tools

The comparison of visualizations of coverage depth between PACVr and three other software tools recovered dissimilar coverage depth distributions. The graphs generated by the tools FastPlast, IOGA, and Circleator were dissimilar among each other and, in the case of FastPlast and IOGA, also dissimilar to PACVr. For the plastid genome of *Nuphar japonica*, FastPlast generated a linear plot of coverage depth that indicated a higher depth in the area corresponding to the IRs than in the large and small SC regions (Fig. 1a). The coverage depth of the IR regions was hereby often larger than 20x and, thus, larger than the manual cap instituted when generating the input files, indicating that the read mapping procedure of FastPlast allows a multiple counting of reads across the input genome. IOGA also generated a linear plot of coverage depth, which indicated a markedly higher coverage depth in an area that approximately corresponds to the SSC compared to other regions of the plastid genome (Fig. 1b); the precise location of this area in relation to the overall genome structure is uncertain, however, as IOGA generates coverage graphs on the concatenation of individual contigs constructed during the assembly process, and these contigs may not be ordered according to their actual position in the genome. Similar to FastPlast, the coverage depth inferred by IOGA surpassed the maximum cap of 20x for certain areas of the genome assembly, indicating a multiple counting of reads. The graph generated by Circleator was the most similar representation of coverage depth compared to the visualization generated by PACVr. Visualized as a circular plot, it indicated areas of reduced coverage depth in the IRs compared to the SC regions and a largely homogeneous coverage depth across the SC regions (Fig. 1c). The precise locations of areas with reduced coverage depth were, however, difficult to determine due to missing references to the quadripartite genome structure and to gene positions. The visualizations of coverage depth for the plastid genome of *Archidasyphyllum excelsum* were also dissimilar among each other as well as to those of PACVr (Additional file 1: Figure S2). Moreover, the coverage graph generated by IOGA for this genome displayed coverage for only ca. 130 kb of the full genome length due to a missing contig (probably an IR) in the assembly product (Additional file 1: Figure S1b).

The dissimilarity of inferred coverage depth distributions between FastPlast, IOGA, Circleator, and PACVr may be the result of different visualization routines among these tools, but may also be impacted by the different plastome assembly procedures employed. The primary function of FastPlast and IOGA is the assembly of complete plastid genomes, with the ability to visualize coverage depth representing a peripheral function. Specifically, FastPlast and IOGA were designed to generate *de novo* plastid genome assemblies from sequence read data, map the sequence reads onto the inferred contigs, and then conduct visualizations of coverage depth to illustrate the assembly results. PACVr and Circleator, by contrast, were designed to visualize the coverage depth of plastid genomes that have been assembled independently of their own functionality. The observed differences in the coverage depth distributions may, therefore, reflect the idiosyncrasies of the genome assembly process as much as the differences in the respective coverage depth calculation and visualization routines. FastPlast, for example, generates plastome contigs via the assembler tools SPAdes [86] and afin [87] in an iterative assembly process, employs sequence coverage as an indicator for assembly accuracy, and calculates coverage depth via the software Jellyfish2 [88] using a fixed 25-mer sliding window. IOGA, by contrast, generates contigs iteratively via SOAPdenovo2 [89], selects the set of contigs with largest scaffold N50 as the new reference assembly, and then calculates coverage depth per base location on this assembly via the script ’bbmap.sh’ of the software BBTools to illustrate the progressive improvement of the assembly output. The resulting visualizations of coverage depth of these software tools are, thus, different in both design and interpretation and can not be directly compared across tools.

## Discussion

### Importance of coverage information in plastid genomics

By visualizing coverage depth in relation to the quadripartite genome structure of plastid genomes and the location of individual genes, PACVr fills the need for a software tool that produces graphically intuitive visualizations for the identification of assembly regions with suboptimal coverage depth. Measuring the coverage depth is critical for the quality assessment of genome assemblies [38]. First, coverage depth is an essential metric for the identification of structural variation, as the depth of sequencing coverage drives the power to detect sequence rearrangements and other structural variants [39]. Generally, greater coverage depth increases the chance that rearrangement endpoints are captured and confirmed by multiple independent reads [43]. This can be particularly relevant in the comparison of complete plastid genomes, which often differ structurally [73], not least in the precise start and end positions of the IR regions [76]. Second, coverage depth is an essential metric for the detection of sequence variation, as genomic regions with exceptionally high [90] or low [91] coverage depths become unreliable for variant calling. In plastid genomics, variant calling can be relevant to identify intra-individual polymorphisms, which are typically generated by the effects of heteroplasmy and common to organelle genomes [92]. Third, de novo assembly algorithms typically operate under the assumption of even coverage depth across the target genome [6, 44, 45], and errors in plastid genome sequences are often correlated with exceptionally high or low coverage depths [29]. The visualization of coverage depth of plastome assemblies, thus, represents an important tool in their quality assessment and should be conducted as early in their bioinformatic processing as possible in order to identify problematic assemblies before proceeding with subsequent analyses. Preferentially, such visualizations should be rapid, easily integrable into automated workflows, and suitable for the evaluation of a large cohort of genome assemblies [38].

### Integration into automated pipelines

Given the demand for high throughput in bioinformatic workflows, individual software tools must be easy to integrate into automated analysis pipelines to be of lasting value for the research community. The integration of plastome assembly and annotation into automated or semi-automated workflows has been proposed and conducted by several investigations [21, 22, 93]. Such workflows are designed to deliver more consistent and repeatable results than the manual administration of individual software tools and provide an ideal platform for the integration of assembly quality tests. However, quality management has so far remained unimplemented in most plastid genome analysis pipelines (but see [21]). In fact, most quality control tools for plastid genome assembly in existence do not provide rapid visualizations of coverage depth. As a result, inaccurate or unsupported plastid genome assemblies may remain undetected and confound subsequent analyses, especially in large, composite investigations that compare hundreds if not thousands of plastid genomes (e.g., [14, 15]). Hence, it is critical to visualize the coverage profile of a plastid genome through an automated, yet user-friendly process that assists in highlighting genomic regions of interest to the researcher [59]. Strong emphasis was, thus, placed on the ability to integrate PACVr into automated bioinformatic pipelines easily. Given this objective, PACVr was customized for and submitted to CRAN, which tests incoming R packages to work on all major operating systems and, thus, ensures these packages to be platform-independent. Similarly, PACVr was designed to enable an operation directly from the Unix shell using CLI arguments, which allows easy integration into automated workflows.

### Importance of open-source software in plastid genomics

Several previously available web-services for visualizing circular plastome maps have become inaccessible over recent years, highlighting the importance of open-source software development in plastid genomics. The development and release of PACVr as an open-source software tool was one of the guiding principles in its development, as this allows other researchers to independently access its source code, customize the software, and extend its functionality. The aim of open-source development is particularly important in the field of plastid genomics, where several previously developed web-services have become inaccessible over recent years. In fact, several interactive web-based tools had been developed to visualize circular chromosomes and their associated metadata, including complete plastid genomes. However, many of these tools are no longer applied because their online interfaces have lost connectivity to the world wide web, and their source code has never been made publicly available. The online platform CARAS [57], for example, offered functionality to annotate and visualize complete plastid genomes and save the results in different output formats, but its web service has been inaccessible since at least February 2017. Similarly, the web platform CGAP [58] offered functionality to generate circular or linear genome maps, annotate assembled plastid genomes, and conduct comparative plastome analysis, but has been inaccessible since at least April 2017. Some of these online services provided installation-free alternatives to the limited number of visualization software tools for plastid genomics [94], and their inaccessibility should be considered a loss for the plant biological research community. Had these services been developed as open-source projects, other researchers would have had the opportunity to continue the maintenance and development of these resources [95]. Open-source development and public accessibility of software tools are, thus, considered critical aspects of bioinformatic software development [96–98]. Consequently, PACVr was developed as an open-source R package that is publicly available via both GitHub and CRAN.

## Conclusions

Coverage depth is often used as an indicator of the quality of a plastid genome assembly. The R package PACVr was designed to visualize coverage depth of plastome assemblies in relation to the circular, quadripartite structure of plastid genomes, the location of individual plastome genes, different window calculation sizes, and user-defined threshold values for coverage depth. PACVr also enables the visual assessment of equality among the IR regions regarding gene presence and synteny. In tests on empirical data, the software successfully visualized the coverage depth and IR equality of complete plastid genomes of different plant lineages, which displayed total plastome sizes between 50 kb and 250 kb. Our evaluations also highlighted that alternative coverage visualization tools for plastid genomes generate incongruent depth visualizations on the same input data, which may be attributable to differences in the visualization process as well as the genome assembly routines. Given its design as an open-source R package with a Unix shell interface, PACVr allows easy integration into bioinformatic pipelines and, thus, provides an important tool for automated quality control in plastid genome sequencing.

## Availability and requirements

**Project name:** PACVr**Project home page:**https://cran.r-project.org/package=PACVr**Operating systems:** Platform independent**Programming language:** R (>= 3.3)**Other requirements:** R packages BiocGenerics, Biostrings, GenomicAlignments, genbankr, optparse; mosdepth (>= 0.2.5)**License:** BSD 3-Clause**Any restrictions to use by non-academics:** none

## Supplementary information

**Additional file 1** Visualizations of coverage depth of the plastid genome of *Archidasyphyllum excelsum* (GenBank accession MH899017) as generated by the software tools FastPlast, IOGA, Circleator, and PACVr. (PDF 8211 kb)

**Additional file 2** Visualizations of coverage depth and IR equality of the plastid genome of *Pelargonium* x *hortorum* (GenBank accession NC_008454) and *Prototheca cutis* (GenBank accession NC_037480). (PDF 5540 kb)

## Data Availability

PACVr is available under the BSD 3-Clause license from CRAN at https://cran.r-project.org/package=PACVr. All datasets analyzed during the present investigation are available from Zenodo at https://zenodo.org/record/3673838.

## Abbreviations

bp: base pairs
CLI: command-line
IR: inverted repeat
kb: kilobases
LSC: large single copy
SSC: small single copy
