# Supplementary material for: PACVr: plastome assembly coverage visualization in R
Source: BMC Bioinformatics. 2020 May 24;21:207. doi: 10.1186/s12859-020-3475-0 (PMC7245912; doi:10.1186/s12859-020-3475-0)

ADDITIONAL FILE 1

PACVr: Plastome Assembly Coverage Visualization in R

Michael Gruenstaeudl[1*] Nils Jenke[2]
[1] Institut für Biologie, Freie Universität Berlin, 14195 Berlin, Germany [2] Institut für Bioinformatik, Freie Universität Berlin, 14195 Berlin, Germany
* m.gruenstaeudl@fu-berlin.de

Fig. S1 Visualizations of coverage depth of the plastid genome of *Archidasyphyllum excelsum* (GenBank accession MH899017) as generated by (**a**) FastPlast, (**b**) IOGA, and (**c**) Circleator. For easier viewing, the size of the individual data points was reduced in the visualization of FastPlast, and the tick mark at 160 kb was removed in the visualization of Circleator.

(a)

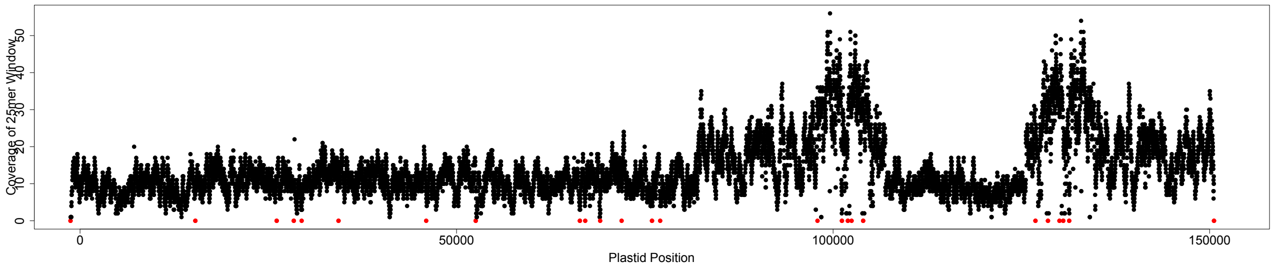
Coverage of 25mer Window
Plastid Position

(b)

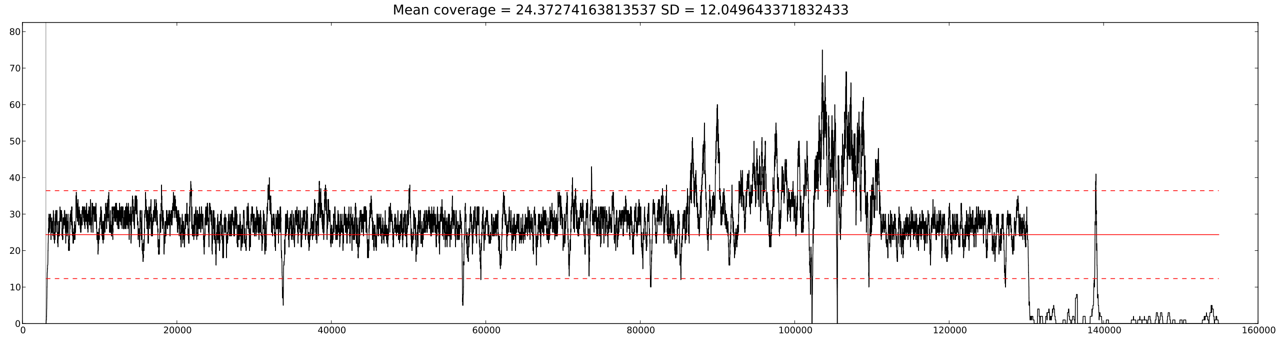
Mean coverage = 24.37274163813537 SD = 12.049643371832433

(c)

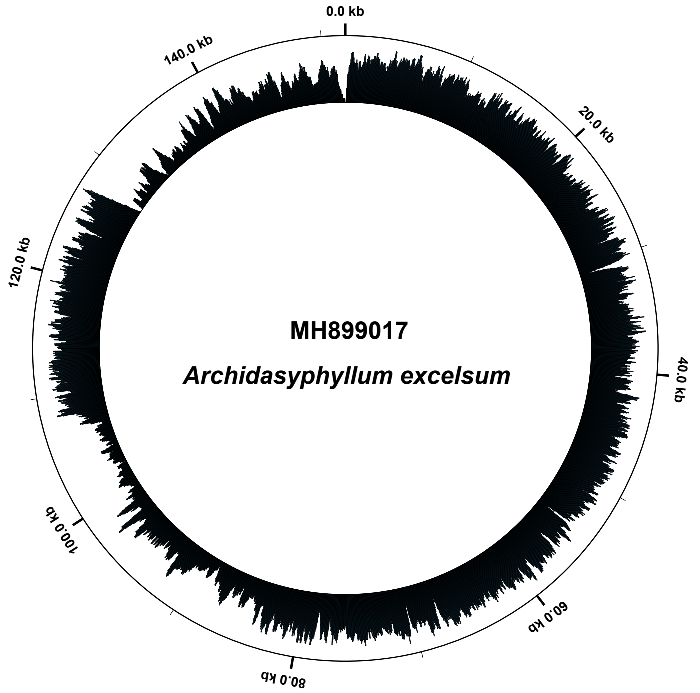
MH899017
Archidasyphyllum excelsum

Fig. S2 Visualization of coverage depth and IR equality of the plastid genome of *Archidasyphyllum excelsum* (GenBank accession MH899017) as generated via PACVr.

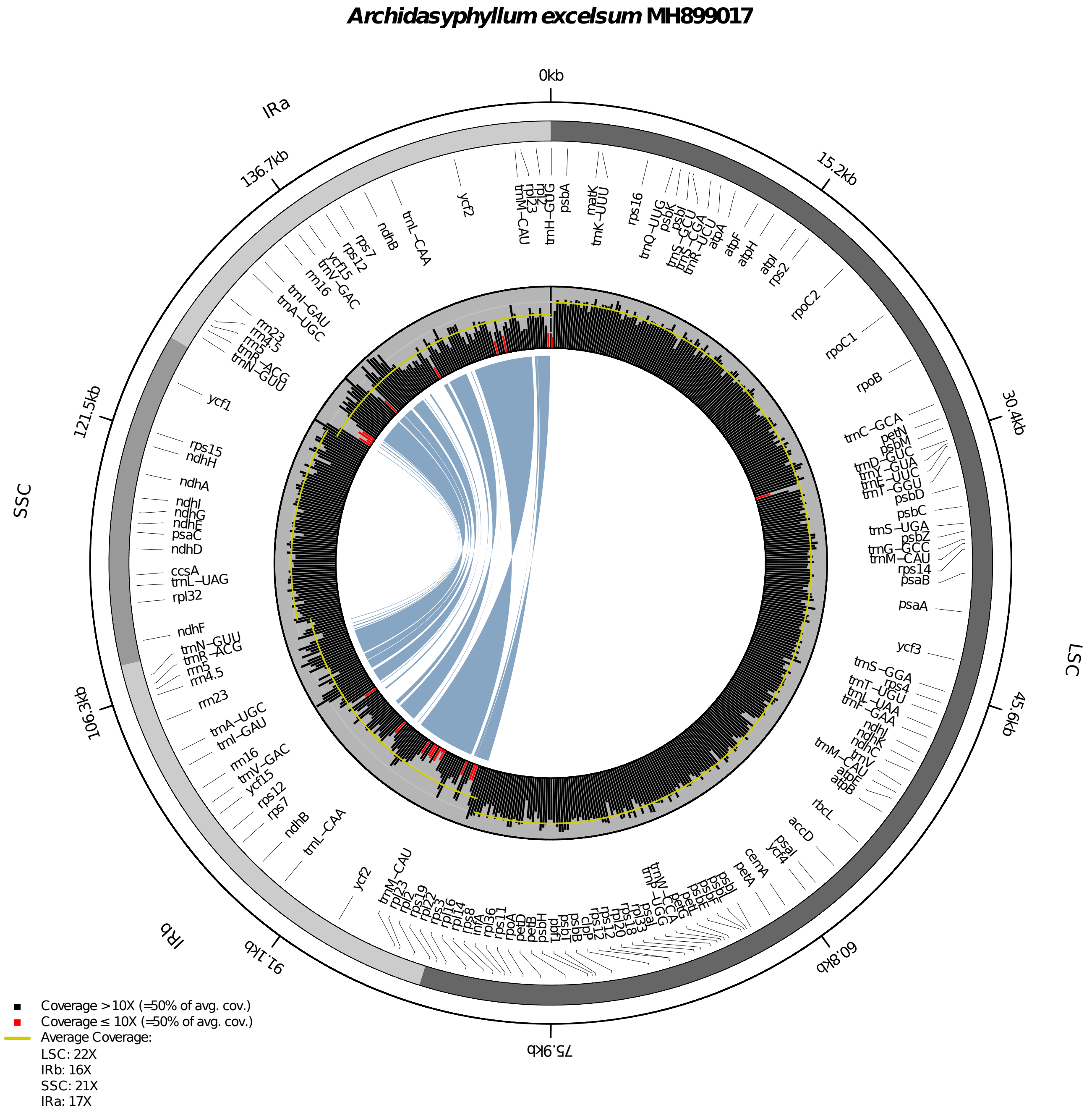

Supplement: Supplementary file 1 — Additional file 1 Visualizations of coverage depth of the plastid genome of Archidasyphyllum excelsum (GenBank accession MH899017) as generated by the software tools FastPlast, IOGA, Circleator, and PACVr. (PDF 8211 kb) [file 12859_2020_3475_MOESM1_ESM.pdf]
