# Supplementary material for: PACVr: plastome assembly coverage visualization in R
Source: BMC Bioinformatics. 2020 May 24;21:207. doi: 10.1186/s12859-020-3475-0 (PMC7245912; doi:10.1186/s12859-020-3475-0)

Additional File 1

PACVr: Plastome Assembly Coverage Visualization in R

Michael Gruenstaeudl[1*] Nils Jenke[2]
[1] Institut für Biologie, Freie Universität Berlin, 14195 Berlin, Germany [2] Institut für Bioinformatik, Freie Universität Berlin, 14195 Berlin, Germany
* m.gruenstaeudl@fu-berlin.de

Fig. S3 Visualization of coverage depth and IR equality of the plastid genome of *Pelargonium x hortorum* (GenBank accession NC_008454), which has a genome size that is substantially larger than the typical size range of angiosperm plastomes.

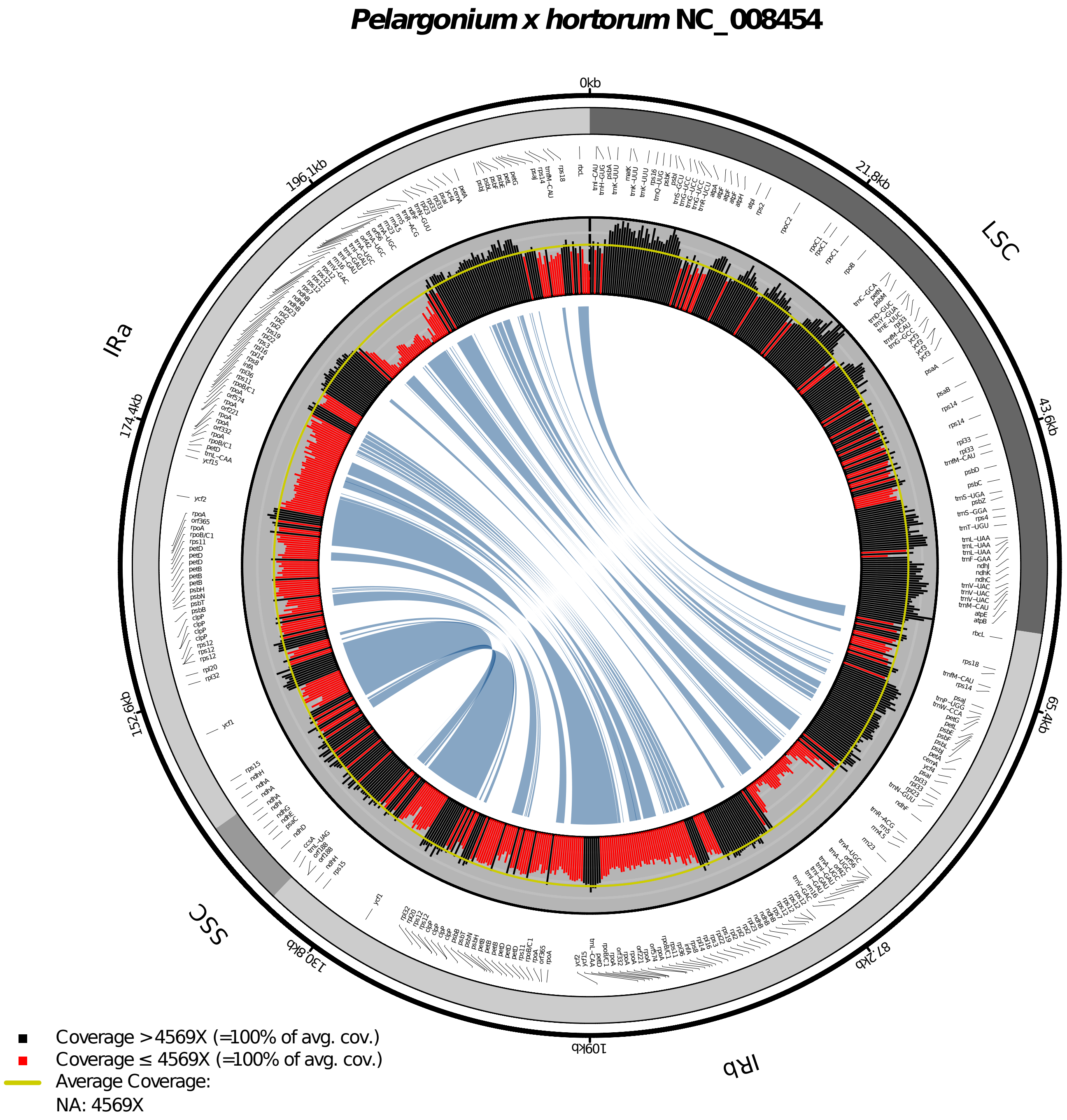

Fig. S4 Visualization of coverage depth and IR equality of the plastid genome of *Prototheca cutis* (GenBank accession NC_037480), which has a genome size that is substantially smaller than the typical size range of green algae plastomes.

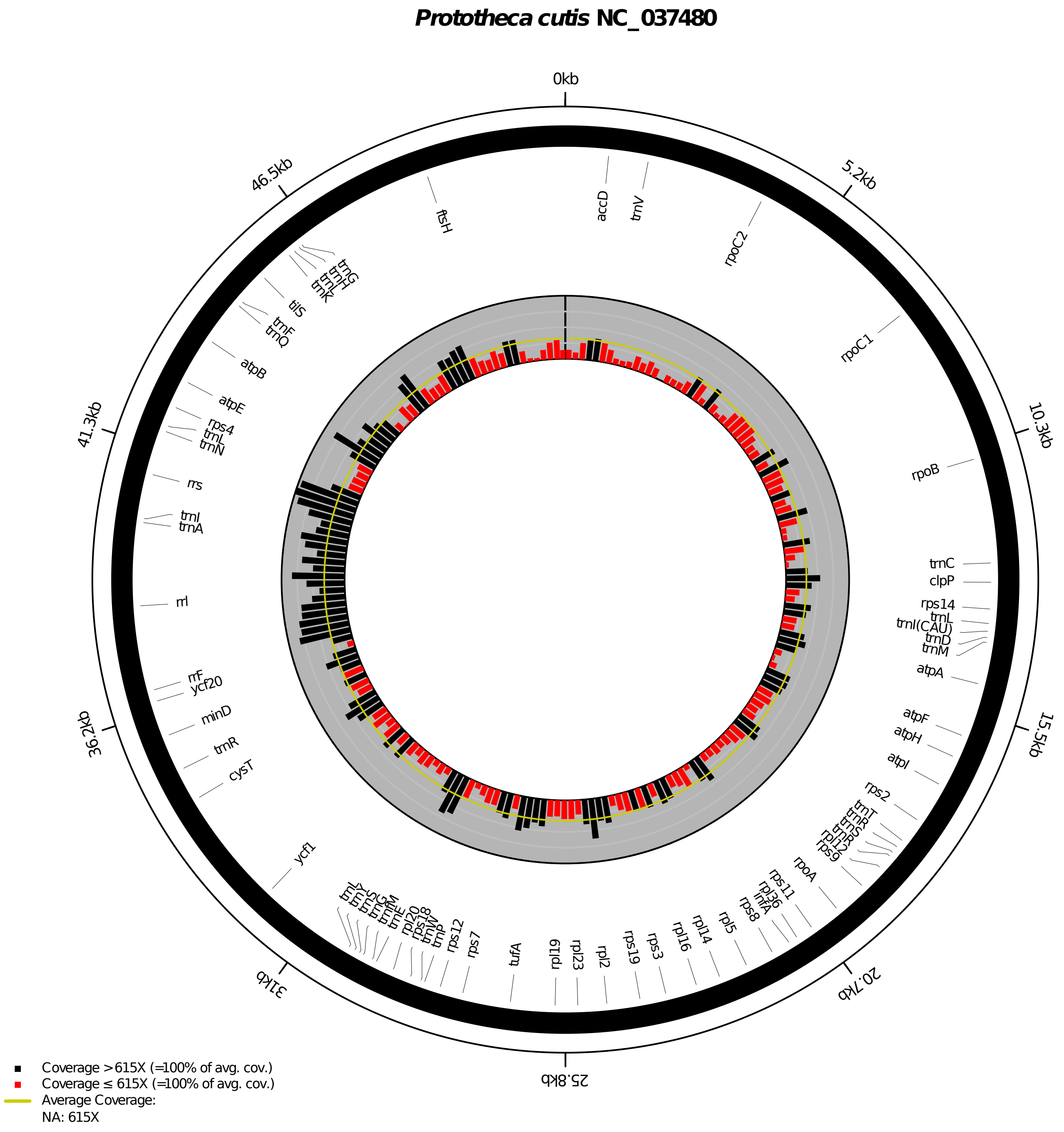

Supplement: Supplementary file 2 — Additional file 2 Visualizations of coverage depth and IR equality of the plastid genome of Pelargonium x hortorum (GenBank accession NC_008454) and Prototheca cutis (GenBank accession NC_037480). (PDF 5540 kb) [file 12859_2020_3475_MOESM2_ESM.pdf]
